# Physical Cue Influences Children’s Empathy for Pain: The Role of Attention Allocation

**DOI:** 10.3389/fpsyg.2018.02378

**Published:** 2018-11-30

**Authors:** Zhiqiang Yan, Meng Pei, Yanjie Su

**Affiliations:** School of Psychological and Cognitive Sciences and Beijing Key Laboratory of Behavior and Mental Health, Peking University, Beijing, China

**Keywords:** child, pain, empathy, eye tracking, physical cue, attentional process

## Abstract

Empathy for pain is evolutionally important and context-dependent. The current study explored the effect of physical cue on 4- to 5-year-old children’s empathy for pain with two experiments. Experiment 1 investigated the effect of valid and invalid physical cue as compared to baseline (without cue) in pain evaluation task (evaluating the pain intensity of a facial expression, *N* = 28). Experiment 2 employed eye-tracking to investigate the attentional process in valid and baseline conditions (evaluating the pain intensity of a body image with an apparently injured arm or leg, *N* = 65). We found the evaluation of pain intensity was the highest in the valid condition, and higher in baseline condition than invalid. As for eye-tracking results, children fixated more quickly, had more fixations and longer total fixation duration in valid-cue condition. Of attention allocation, compared with baseline condition, children fixated on arm/leg more quickly, more frequently and for longer time in valid condition. Additionally, eye-tracking results were significantly related to their evaluation of pain intensity.

## Introduction

Pain, as a sublethal injury, could threat individual’s survival and racial reproduction (Walters, 1994). Thus, evaluation of other’s pain intensity would be a key skill in social animals. The ability of empathy is closely related to the evaluation of other’s pain (Goubert et al., 2005; Decety and Svetlova, 2012), but also gives rise to the problem of faking pain to one’s own advantage. Fortunately, previous research has suggested us that both pain (Steinkopf, 2016) and empathy (Melloni et al., 2014) are context-dependent, meaning that people could avoid showing empathy for other’s fake pain with the help of contextual cue. Evolutionally, we would expect children could take advantage of contextual cue (especially physical cue) to show empathy for pain. After all, it is essential to individual and racial reproduction (Williams, 2002; de Waal, 2008; Williams and Craig, 2016).

According to the International Association for the Study of Pain (IAPS), pain is an unpleasant sensory and emotional experience associated with actual or potential body damage, or described in terms of such damage (Merskey and Bogduk, 1994; Williams and Craig, 2016). Evolutionary psychology suggests us that pain has two facets of functions. First, to draw the observer’s attention for prompt “fight or flight” response. Second, to warn the observer of threats, evoking empathic and pro-social behaviors (Williams, 2002; Goubert et al., 2005). A number of studies have shown that perception and genuineness judgment of other’s pain were closely linked to empathy (Batson, 1991; Danziger et al., 2006; Decety et al., 2016). For example, people scoring higher in empathy would exhibit higher pain-related brain activity (Singer et al., 2004; Jackson et al., 2005), and observers’ brain activity was in turn related to the rated intensity of pain presented (Saarela et al., 2007). Non-human animals also would show empathy for their conspecifics’ pain (de Waal and Preston, 2017; Meyza et al., 2017; Meyza and Knapska, 2018). Empathy is often considered as a major motivator driving prosociality (Jensen, 2016), and is a main factor that urges people to behave altruistically (Gluth and Fontanesi, 2016; Hein et al., 2016). Pharmacology research also found that a common physical painkiller, acetaminophen (paracetamol), can reduce empathy for another’s pain (Mischkowski et al., 2016), which suggests us that empathy and pain could share the same physiological mechanisms.

However, people are poor at detecting deception and fake pain from capable fakers (Leach et al., 2009; Bartlett et al., 2014), and even would show a higher evaluation for fake pain than for true pain (Poole and Craig, 1992). Therefore, faking pain is reinforced by high return and low risk. Because empathy for pain has a cost (Loggia et al., 2008), judgment for genuineness is necessary (Hadjistavropoulos et al., 2011). Many studies have showed that empathy for pain is highly context-dependent (Gonzalez-Liencres et al., 2013; Melloni et al., 2014). Thus, people would take advantage of contextual cues to make decisions, as they could provide us explainable information and would benefit causal reasoning (Steinkopf, 2016).

In daily life, contextual cues would consist primarily of two forms: physical cues (e.g., tools, such as needle) (Sun et al., 2017), and social cues (e.g., facial expressions) (Grynberg and Maurage, 2014). Recent functional magnetic resonance imaging (fMRI) findings suggest that physical cues and social cues activate common brain regions (Singer et al., 2004; Jackson et al., 2005; Eisenberger, 2012). As we mentioned before, if empathy for pain is important in evolution, the ability to make use of contextual cues to make a proper empathic response for pain would appear early in life. There are some research investigating how social cues influence children’s empathy for pain. Deyo et al. (2004) found that 3- and 12-year-old children could detect pain in others and assess pain intensity indexed by the cue of facial expression. Another study found that 4- to 6- years old children would use physical cue to evaluate other’s pain intensity (Grégoire et al., 2016; Yan et al., 2017). Also, it is found that physical cues would influence people’s empathy for pain (Wu et al., 2017). But, during the process to communicate pain, “wounded person” may have an advantage from manipulating the observer. In the other words, observer is always in a passive position (Steinkopf, 2016). Additionally, comparing with social cues such as facial expression of pain and verbal report, physical cues such as an open wound would induce a robust and stronger reaction (Vachon-Presseau et al., 2011).

In this paper, we took a fresh look at the relationship between physical cue and empathy for pain in children. To measure children’s attentional process comprehensively, we combined pain evaluation task and eye-tracking. As previous research suggests, pain as a signal would first induce self-oriented emotion and activate threat detection system, and in the later stage of attentional processing the perceived threat associated with pain would be influenced by personal trait or the context (Goubert et al., 2005, 2009). The attentional process is usually divided into two stages: attention orientation and attention maintenance (Vervoort et al., 2013; Todd et al., 2016), and previous research showed that empathic response for pain would be different in these two stages (Yan et al., 2016). This formed the basis for the current study to investigate the dynamics of the attentional process.

### Current Study

We attempted to answer the following two questions in this paper: (1) Would physical cues influence children’s empathy for pain? (2) What was the relationship between children’s empathy for pain and attentional processing? Specifically, we used two types of physical cues: pictures of tools that could cause injury as “valid” cues, which were typically associated with true or genuine pain, and pictures of tool-shaped soft toys as “invalid” cues, which were indicative of fake or deceptive pain.

The current study employed two experiments to answer these two questions. In Experiment 1, 28 participants were asked to evaluate other’s pain intensity and estimate the genuineness based on other’s facial expression with a physical cue (valid or invalid) or with no cue (baseline). In Experiment 2, 65 participants viewed other’s body (painful facial expression presented along with an injured or intact leg or arm) and were asked to evaluate other’s pain intensity and judge the genuineness. Eye tracking measures were recorded in Experiment 2. We predicted physical cues would influence children’s evaluation of pain intensity, and valid physical cue would increase children’s empathy for pain, invalid physical cue causing the opposite. As for attentional process, due to the features of physical cue we used in this study, we expect that valid physical cue would induce a quicker attention orientation, but only attention maintenance could predict children’s evaluation of pain intensity.

## Experiment 1: Physical Cue and Empathy for Pain in Children

### Participants

G^∗^Power (version 3.1, Faul et al., 2009) showed that a sample size of 28 was required for a power of 80% to detect an effect of *f* = 0.25 at α = 0.05. Twenty-eight 4- to 5-year-old children were recruited from a local kindergarten. According to the kindergarten’s official records, these children were normally developing and showed no signs of mental disease. This experiment was approved by the Ethics Committee of the School of Psychological and Cognitive Sciences at Peking University. In accordance with the Declaration of Helsinki, we provided parents of each participant with a written description of the experiment before it began, and all parents stated in written informed consent that they allowed their child to participate. Finally, data from all 28 children (*M*_age_ = 65.99 months, *SD*_age_ = 7.16; 15 males) were analyzed. Gender was equally distributed within this sample [*χ^2^* (1, *N* = 28) = 1.53, *p* > 0.05]. According to previous meta-analyses (Lamm et al., 2011; Yan and Su, 2018), there was no gender difference in empathy, thus the variable of gender was not further analyzed.

### Design and Material

Different physical cues would be used to test how physical cues influenced children’s evaluation of pain. Therefore, a one-factor (physical cue: valid, invalid and baseline) experimental design was planned.

Facial expressions of pain were taken from Yan et al. (2017). Each image was 200 × 250 pixels, or 7.1 × 8.8 cm in size, and luminance was controlled. The faces were taken from a previous study (Yan et al., 2017), in which all faces were rated on valence, arousal and emotional intensity. The mean ratings for the eight faces used here were 2.87 out of 9, 5.49 out of 9, 4.53 out of 6 respectively. The pictures of valid physical cues (e.g., a hammer hitting a hand) was collected from Jackson et al. (2005). With Photoshop (version CS6), we replaced valid cues with invalid ones (e.g., hammer-like balloon on a hand) and cartoonized them as Gu and Han (2007). Next, we invited twenty-two undergraduates majoring in psychology to evaluate these physical cue pictures on 3 dimensions with Likert scales. They were instructed to “rate how the person in the picture might feel, with respect to emotional valence: 1 = clearly unpleasant to 9 = clearly pleasant; and arousal: 1 = highly relaxed to 9 = high level of arousal; and pain intensity: 1 = not pain to 9 = very intense pain.” Valid and invalid cues differed on all 3 dimensions and statistics are shown in Table 1. Finally, eight facial expressions of pain, eight valid physical cue pictures and eight invalid physical cue pictures were selected.

**Table 1:** Rating on the physical cues.

	Valid	Invalid	Statistics		
			
	*M(SD)*	*M(SD)*	*t*(21)	*p*	Cohen’s *d*
Valence	1.48(0.92)	2.89(1.57)	-4.22	<0.001	-1.09
Arousal	7.43(1.27)	4.92(1.39)	7.21	<0.001	1.89
Pain intensity	7.99(1.00)	4.40(1.57)	9.66	<0.001	2.73

### Apparatus and Measures

The experimental task was a pain evaluation task, adapted from previous researches (Deyo et al., 2004; Yan et al., 2017), and programmed with Python (version 3.6.3). In order to make the evaluation more manageable for children, we used the FACES scale, ranging from 0 to 10 (Wong and Baker, 1988), which had been validated by many studies (Yu et al., 2009; Garra et al., 2013). Participants were told to evaluate the intensity of pain, from 1 (no pain) to 6 (very intense pain), and their ratings would be transformed to 0-2-4-6-8-10 for analysis. After each trial, they would decide whether this person was showing “true” or “fake” pain, or they were “unsure” about this. Percentage of the three answers would be analyzed. We defined fake pain as a state which involved one intentionally pretending to be suffering from pain. Each of eight facial expressions of pain would be presented in three trials with a valid cue, an invalid cue and baseline, respectively, yielding a session of 24 trials. Always baseline block present first, and valid or invalid physical cue later.

### Procedure

**FIGURE 1:**
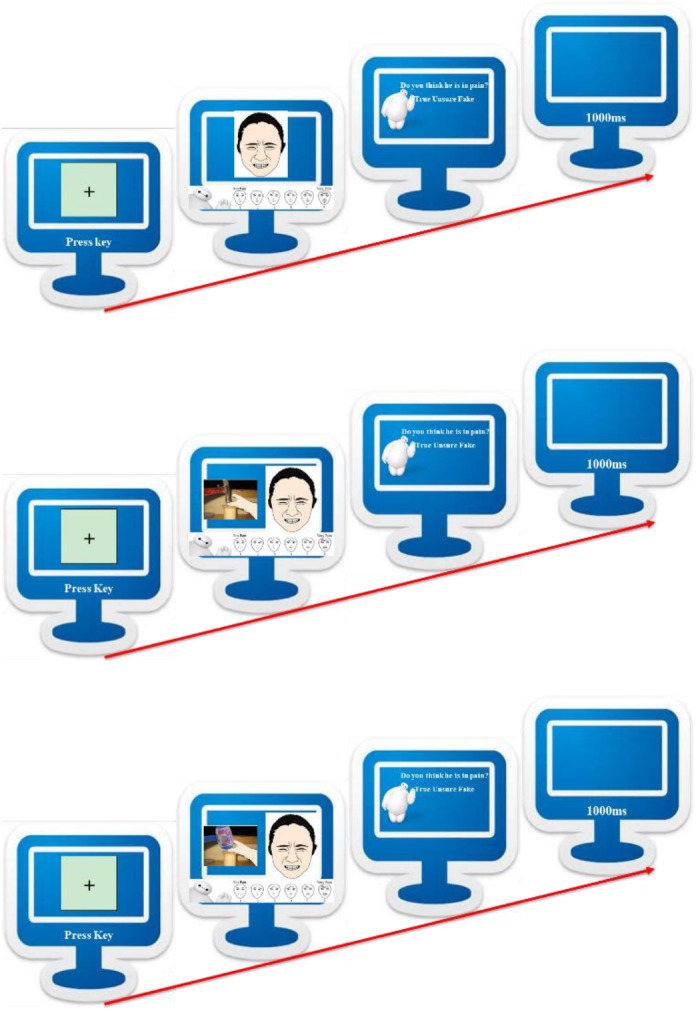
Pain evaluation task diagram, baseline condition **(top)**, valid-cue condition **(middle)**, and invalid-cue condition **(bottom).**

Upon arrival, children were invited to complete the pain evaluation task (see Figure 1). They were instructed that the screen would first present a fixation cross, then simultaneously a physical cue (only in the valid- and invalid-cue conditions), a facial expression of pain and a FACES scale. At the same time, experimenter would give instruction, “this person’s hand was hit by the hammer, and he showed this facial expression,” and children would be asked how intense the pain was and whether the pain was true, fake or they were unsure. There was no time limit and the next trial would begin 1 s after the participant made a genuineness judgment. Upon completion, they would get a toy for participation.

### Results

**FIGURE 2:**
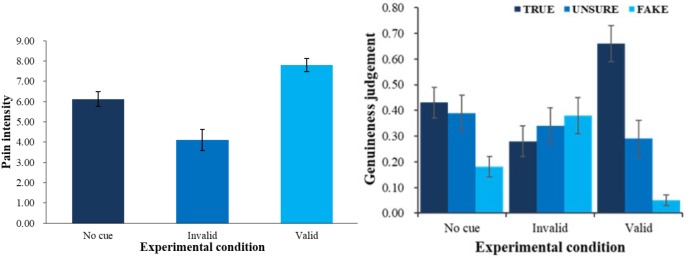
Pain intensity **(left)** and genuineness judgment **(right)** with different physical cue. Error bars show standard errors in this and the following figures.

First, we did a one-factor repeated measures ANOVA on evaluated pain intensity. There was a main effect of condition *F*(2,54) = 29.05, *p* < 0.001, η_p_^2^ = 0.52. Follow-up pairwise comparisons showed that evaluated intensity in valid-cue condition (*M* = 7.80, *SE* = 0.32) was higher than baseline condition (*M* = 6.13, *SE* = 0.35) and invalid-cue condition (*M* = 4.11, *SE* = 0.53), and baseline condition yielded higher evaluated intensity than invalid-cue condition (see left panel of Figure 2, all *ps* < 0.01). All pairwise comparisons in the current article were done with Bonferroni correction.

Second, a 3 (physical cue: valid, invalid and baseline) × 3 (genuineness judgment: true, unsure, fake) ANOVA was employed (see right panel of Figure 2) to analyze the patterns of judgment in different conditions. There was an interaction between physical cues and genuineness judgment *F*(4,108) = 12.64, *p* < 0.001, η_p_^2^ = 0.32. Simple effect analysis (all *ps* < 0.05) showed that in the baseline condition, children were more likely to choose true (*M* = 0.43, *SE* = 0.06) and unsure (*M* = 0.39, *SE* = 0.07) than fake (*M* = 0.18, *SE* = 0.04); in the valid-cue condition, they were more likely to judge the pain as true (*M* = 0.66, *SE* = 0.07) than fake (*M* = 0.05, *SE* = 0.02) and unsure (*M* = 0.29, *SE* = 0.07), and unsure than fake. We also found a main effect of genuineness judgment *F*(2,54) = 4.36, *p* < 0.05, η_p_^2^ = 0.14. Follow-up pairwise comparisons showed that overall, children were more likely to judge the pain as true (*M* = 0.45, *SE* = 0.05) than fake (*M* = 0.20, *SE* = 0.04, *p* < 0.01).

### Discussion

Experiment 1 found that valid physical cue would bring up children’s evaluation of other’s pain intensity, and invalid physical cue would lower down the evaluation. We also found that children would more likely perceive other’s pain as true than as fake in valid-cue condition, whereas there was a trend for the opposite result in invalid-cue condition.

These results in large agree with our hypothesis. In line with previous research (Sun et al., 2017), participants would think that painful facial expression coupled with a valid physical cue would be more likely to be true. Although children in this experiment could distinguish different physical cue would cause different intensity of pain, they still tend to think that these people are not fake pain. As far as we concerned, this result would be attributed to the facial expression of pain. Albeit painful facial expression would couple with valid or invalid physical cue, the result is the painful facial expression. It suggested that children might be more influenced by facial expression. Indeed, facial expression of pain is one of the most salient social cues of other’s pain (Kappesser and Williams, 2002; Williams, 2002). When we attempt to evaluate other’s pain intensity, facial expression would be the best predictor (Igier et al., 2014).

Previous research of empathy for pain mainly focused on participants expectation of pain (Lobanov et al., 2014), and usually would not involve actually presenting painful expression. By simultaneously presenting physical cue and facial expression of pain, we give participants the full story with cause and effect. Therefore, even if a physical cue could modulate children’s evaluation of pain, it still could not counteract the effect of facial expression of pain entirely.

In Experiment 1, we found that physical cue could modulate children’s evaluation of other’s pain intensity. But, we didn’t know what cognitive processes were involved. Therefore, to give a full image of children’s cognitive processes during evaluation of other’s pain, Experiment 2 was done. In order to improve the ecological validity of materials, we presented physical injury and facial expression in one uncropped picture of a character. Due to the difficulty of recognizing invalid physical cue in daily life, thus we only include baseline and valid-cue conditions in Experiment 2.

## Experiment 2: Physical Cue and Attentional Allocation in Children’s Empathy

### Participants

G^∗^Power (version 3.1, Faul et al., 2009) showed that a sample size of 62 was required for a power of 80% to detect an effect of *f* = 0.25 at α = 0.05. Seventy-four 4- and 5-year-old children who did not take part in Experiment 1 were recruited from a local kindergarten. According to the kindergarten’s official records, these children were normally developing and showed no signs of mental disease. This experiment was approved by the Ethics Committee of the School of Psychological and Cognitive Sciences at Peking University. In accordance with the Declaration of Helsinki, we provided parents of each participant with a written description of the experiment before it began, and all parents stated in written informed consent that they allowed their child to participate. Nine were excluded because they failed to meet the same data quality criteria (e.g., eye movements tracked < 50% of total viewing time in task, see Frank et al., 2009) and results a sample of 65 (*M*_age_ = 57.62 months, *SD*_age_ = 3.08; 35 males) were reported, but this exclusion did not affect the nature of the results in terms of significance and directionality. Gender was equally distributed within this sample [*χ^2^* (1, *N* = 65) = 0.39, *p* > 0.05]. According to previous meta-analyses (Lamm et al., 2011; Yan and Su, 2018), there was no gender difference in empathy, thus the variable of gender was not further analyzed.

### Design and Material

This experiment employed a 2-level (physical cue: valid, baseline) within-subject experimental design for pain evaluation, and a 2 (physical cue: valid, baseline) × 2 (body part: face, arm/leg) design with eye-tracking indices as dependent variables. A valid cue was a red dot on the character’s arm or leg where he covered the skin with one hand or leg, and in the baseline condition, there was no red dot on the limb and the character just covered it.

A set of 24 pictures were selected from internet and edited by Photoshop (version CS6). Next, we invited 24 undergraduates majoring in psychology to evaluate these pictures on 3 dimensions with Likert scales, as well as experiment one (see Table 2). Finally, ten valid physical cue pictures and ten invalid physical cue pictures with the same facial expression were selected randomly.

**Table 2:** Experiment material rating results.

	Valid	Baseline	Statistics		
			
	*M(SD)*	*M(SD)*	*t*(23)	*p*	Cohen’s *d*
Valence	2.68(1.93)	2.47(1.30)	1.10	>0.05	0.46
Arousal	4.77(1.80)	3.92(1.59)	4.34	<0.001	1.81
Pain intensity	5.81(1.50)	4.41(1.90)	5.18	<0.001	2.16

### Apparatus and Measures

A Tobii T120 Eye-tracker (Tobii Technology AB, Sweden) was used to show the stimuli (sample rate = 120 Hz, resolution = 1024 × 768). The experiment was programmed and analyzed with Tobii Studio. Children were seated about 60 cm from the computer screen and a chin rest was used to restrict head movement. A gaze that remained stable within a 35-pixel radius and lasted at least 100 ms on a defined area of interest (AOI) would count as fixation to that AOI (Yang et al., 2012; Vervoort et al., 2013; Yan et al., 2017).

As in previous research (Yang et al., 2012; Vervoort et al., 2013; Yan et al., 2017), three eye tracking indices were used. The first one, time to first fixation, was defined as the time it took following the onset of a picture set to first fixation on a specific AOI (i.e., facial expression or arm/leg). The second, fixation count, was defined as the total fixation counts that a participant made within the rectangular picture containing a particular body area as a stimulus. The third, total fixation duration, was defined as the total duration of time in which a participant’s gaze remained fixated within the boundaries of a particular body area.

### Procedure

The same as experiment one. Eye movement was recorded through the experiment, and we divided the picture into 2 parts (sub-AOIs) for further analysis: face and arm/leg. Upon completion, they would get a toy for participation.

### Results

**FIGURE 3:**
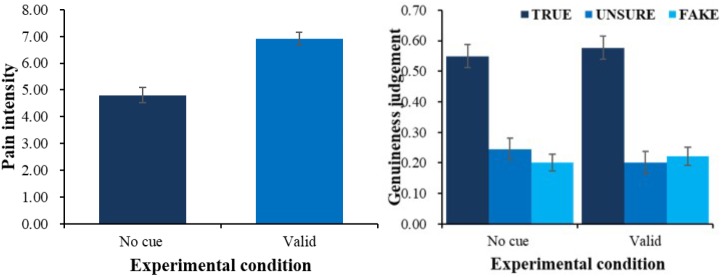
Pain intensity **(left)** and genuineness judgment **(right)** of different physical cue.

Experiment 2 replicated Experiment 1’s results (see Figure 3). On evaluation, there was a main effect of physical cue, *F*(1,64) = 58.44, *p* < 0.001, η_p_^2^ = 0.48. Evaluation of pain in valid-cue condition (*M* = 6.91, *SE* = 0.23) was higher than in baseline (*M* = 4.80, *SE* = 0.29, *p* < 0.001).

A 2 (physical cue: valid, baseline) × 3 (genuineness judgment: true, unsure, fake) ANOVA on proportion of choice also showed that there was an interaction between physical cue and genuineness judgment *F*(2,128) = 5.10, *p* < 0.01, η_p_^2^ = 0.07. Simple effect analysis suggested that compared with baseline condition (*M* = 0.54, *SE* = 0.04), proportion of choosing true (*M* = 0.58, *SE* = 0.04, *p* = 0.08) was higher in the valid-cue condition, and in both conditions (all *ps* < 0.01) true was more frequently chosen than unsure (*M* = 0.20, *SE* = 0.03) and fake (*M* = 0.22, *SE* = 0.03). Additionally, there was a main effect of genuineness judgment *F*(2,128) = 23.51, *p* < 0.001, η_p_^2^ = 0.27, follow-up pairwise comparisons showed that participant chose true (*M* = 0.56, *SE* = 0.04) more than unsure (*M* = 0.23, *SE* = 0.04, *p* < 0.001) and fake (*M* = 0.21, *SE* = 0.03, *p* < 0.001).

**FIGURE 4:**
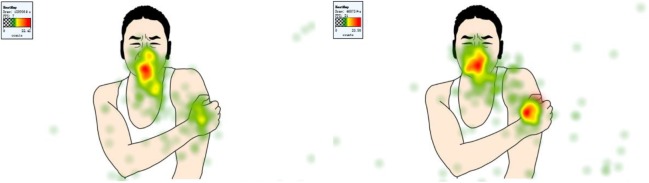
Eye tracking heat map in baseline condition **(left)** and valid-cue condition **(right).**

**FIGURE 5:**
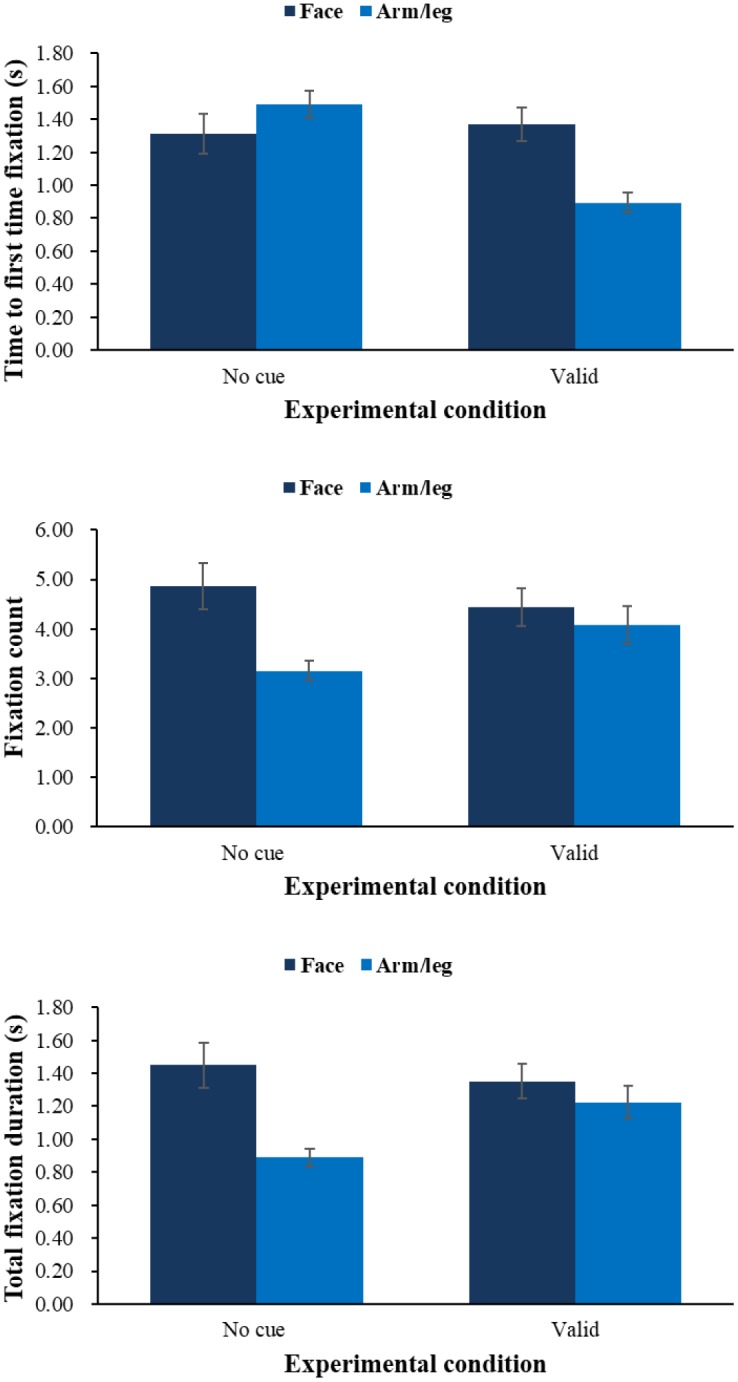
Means of time to first fixation **(top)**, fixation count **(middle)**, and total fixation duration **(bottom)** during pain evaluation task by condition of physical cue.

In order to investigate the effects of physical cue on attention, a series of 2 (physical cue: valid, baseline) × 2 (body part: face, arm/leg) ANOVAs on 3 eye-tracking indices was employed. Sample stimuli are shown in Figure 4 and results in Figure 5.

On time to first fixation, results showed that there was an interaction effect between physical cue and body part *F*(1,64) = 34.09, *p* < 0.001, η_p_^2^ = 0.35. Simple effect analysis showed that children would fixate onto arm/leg (*M* = 0.89 s, *SE* = 0.06) more quickly than facial expression (*M* = 1.37 s, *SE* = 0.10, *p* < 0.001) in the valid-cue condition, and children would fixate onto arm/leg more quickly in the valid physical condition than no-cue condition (*M* = 1.49 s, *SE* = 0.08, *p* < 0.001). And physical cue had a main effect *F*(1,64) = 17.33, *p* < 0.01, η_p_^2^ = 0.21, showing that children would have a shorter time to first fixation in the valid-cue condition (*M* = 1.13 s, *SE* = 0.07) than no-cue condition (*M* = 1.40 s, *SE* = 0.09, *p* < 0.001). No other effects were found. On fixation count, we found an interaction between physical cue and body part *F*(1,64) = 16.52, *p* < 0.001, η_p_^2^ = 0.21. Simple effect analysis showed that children would fixate on facial expression (*M* = 4.86, *SE* = 0.48) more times than arm/leg (*M* = 3.15, *SE* = 0.20, *p* < 0.001) in no-cue condition, and compared with no-cue condition (*M* = 3.15, *SE* = 0.20) children would have more fixation counts on arm/leg in the valid-cue condition (*M* = 4.08, *SE* = 0.38, *p* < 0.05), and compared with valid-cue condition (*M* = 4.44, *SE* = 0.38) children would have more fixation counts on facial expression in the no-cue condition (*M* = 4.86, *SE* = 0.48). There were main effects of physical cue *F*(1,64) = 3.39, *p* = 0.07, η_p_^2^ = 0.05 and body part *F*(1,64) = 23.70, *p* < 0.001, η_p_^2^ = 0.27. Follow-up pairwise comparisons showed that children would have more fixation counts in the valid-cue condition (*p* = 0.07), and children would fixate on facial expression (*M* = 4.65, *SE* = 0.42) more times than arm/leg (*M* = 3.61, *SE* = 0.28, *p* < 0.001). On total fixation duration, we found the same pattern of results as with fixation count. There was an interaction between physical cue and body part *F*(1,64) = 19.16, *p* < 0.001, η_p_^2^ = 0.23, simple effect analysis (all *ps* < 0.05) showed that children would fixate on arm/leg longer in the valid-cue condition (*M* = 1.23 s, *SE* = 0.10) than no-cue condition (*M* = 0.89 s, *SE* = 0.06), and fixate on facial expression (*M* = 1.45 s, *SE* = 0.14) longer than arm/leg (*M* = 0.89 s, *SE* = 0.06) in the no-cue condition. There were also main effects of physical cue *F*(1,64) = 9.46, *p* < 0.01, η_p_^2^ = 0.13 and body part *F*(1,64) = 18.68, *p* < 0.001, η_p_^2^ = 0.23. Follow-up pairwise comparisons showed that children would fixate longer in the valid-cue condition (*M* = 1.29 s, *SE* = 0.09) than no-cue condition (*M* = 1.17 s, *SE* = 0.09, *p* < 0.01), and fixate on facial expression (*M* = 1.40 s, *SE* = 0.12) longer than arm/leg (*M* = 1.06 s, *SE* = 0.07, *p* < 0.001).

Finally, we explored the relationship between children’s eye tracking indices and the evaluation of pain intensity (see Table 3). In order to create measures combining pain intensity and genuineness judgment, we multiplied each child’s mean evaluation of pain intensity by the proportion of judgment as true, and termed the product as true pain intensity. The same was done with the proportion of judgment as fake, to calculate fake pain intensity. Results showed that children’s evaluation of pain intensity was positively related to their fixation count and total fixation duration on facial expression in the baseline condition, and marginally positively related to fixation count on arm/leg in the valid-cue condition. Children’s evaluation of true pain intensity was positively related to their fixation count and total fixation duration on face in both conditions, and positively related to fixation count and total fixation duration on body part of arm/leg in the valid-cue condition. Evaluation of fake pain intensity was not related to any eye-tracking index.

**Table 3:** Correlation between eye tracking index and the evaluation of pain intensity.

		Baseline			Valid	
		
	Pain intensity	True pain intensity	Fake pain intensity	Pain intensity	True pain intensity	Fake pain intensity
faceTFF	-0.06	0.01	0.08	0.09	0.04	0.06
arm/legTFF	0.15	0.24^+^	-0.04	0.07	0.10	-0.11
faceFC	0.36**	0.32**	-0.03	0.29*	0.26*	-0.07
arm/legFC	0.01	0.14	-0.15	0.22+	0.29*	-0.19
faceTFD	0.38**	0.32**	-0.01	0.28*	0.29*	-0.06
arm/legTFD	0.01	0.07	-0.14	0.20	0.24^+^	-0.18

*^+^0.05 < p < 0.10, ^∗^p < 0.05, ^∗∗^p < 0.01.*

### Discussion

Experiment 2 not only replicated the results of Experiment 1, but also found that children’s evaluation of pain was closely related to their degree of attentional allocated. First, children would fixate quickly, for more times and longer in the valid-cue condition; second, children’s fixation count of face and total fixation duration of face was positively correlated with children’s evaluation of pain and true pain intensity in both two conditions, and we additionally found that fixation count and total fixation duration on arm/leg was correlated with children’s evaluation of true pain intensity in the valid-cue condition.

The relationship between pain and attentional process is a classical question. Dot-probe paradigm would be the most popular paradigm to investigate the relationship between attention and pain (Roelofs et al., 2003), and in this way research has consistently found an effect of attentional bias toward pain-related information, whether the materials were words (Yang et al., 2012) or pictures (Vervoort et al., 2013; Yan et al., 2016). In line with these studies, we also found that physical cue would influence children’s attentional process. Specifically, we have found that children would have a shorter time to first fixation in valid-cue condition, which could be expected as the physical cues in our experiment were salient. And we would be wondering whether children’s attention allocation correlated with their empathy for pain. Although we did not find any correlation between children’s time to first fixation to face or arm/leg and evaluation of pain in valid-cue condition, we found that fixation count and total fixation duration were significantly related to children’s empathy for pain, and attention to arm/leg part correlated with true pain intensity only in valid-cue condition. That is to say, children’s automatic attentional process, which was influenced by physical cue, was not correlated with empathy for pain. Yet fixation count and total fixation duration correlated with empathy for pain, meaning that attention maintenance was more closely associated with empathy for pain. Note that fixation count and total fixation duration are indices of the later stage of attentional process, which is involved in higher cognitive processes. Previous research found that participants who were higher in empathy would pay more attention when discriminating facial expressions (Choi and Watanuki, 2014) and would have a longer fixation duration on emotional stimuli (Cowan et al., 2014). Our own research (Yan et al., 2016) found similar results and proposed that attention maintenance would closely correlate with participants’ empathy. Experiment 2 suggested that when children tried to evaluate other’s pain intensity, especially true pain intensity, they would seek peripheral information and take advantage of any contextual cue available (Craig, 2015).

## General Discussion

To sum up, the current study did two experiments and found that physical cues would affect children’s empathy for pain, and it played this role by influencing children’s attentional allocation, especially the later stage of attentional process. These findings showed us a detailed image for relationship between physical cues and children’s empathy for pain.

### The Relationship Between Physical Cues and Empathy for Pain

Contextual cue (Craig, 2015) would be an important determinant to how we evaluate the intensity and judge the genuineness of other’s pain. Both experiments in the current study, provided support for the influence of physical cue to children’s evaluation of pain. It should be noted that the cues in Experiments 1 and 2 differs a little in nature, since they served different purposes. In Experiment 1, we wanted to introduce an invalid cue and a tool-like toy would be the best as it was difficult to present a wound-like mark that could be easily recognized as “invalid” by children. In Experiment 2, a wound cue would lend itself very well to eye-tracking technique for studying attention. In daily life, a wound is a more common and more direct sign of pain than a tool that might cause harm, and in this sense, Experiment 2 had higher ecological validity. Due to the same facial expression of pain, if children try to evaluate other’s pain intensity and make genuineness judgment, they need to take advantage of contextual cue (e.g., physical cue). Sun et al. (2017) have used ERP methodology to test how adults differentiated true (being pricked by a needle) from fake (being touched by a swab) pain for a proper empathic response, and they found that adults would react fast and have a higher accuracy rate in the true condition. Our study also found that early in life, children could use physical cue to evaluate other’s pain and make genuineness judgment. Children made more “true” judgments than other types in valid-cue condition and showed a trend to make more “fake” judgments in invalid-cue condition. Song et al. (2016) also found that young children could discriminate genuine from fake smiles. In all, the literature indicated that children could understand what a fake or pretended emotional state meant, yet it would be better in future studies to ensure this by explicitly asking them about it.

### The Relationship Between Physical Cues and Attentional Process

The influence of physical cue on children’s evaluation of pain could be interpreted in terms of its influence on attention allocation. As previous research found, other’s painful facial expression would evoke observer’s empathy for pain (Goubert et al., 2005; Grynberg and Maurage, 2014), which was in turn associated with pain-related stimuli (Yan et al., 2017).

Many studies have indicated that evaluation of other’s pain was closely related to the process of attention, especially the late stage. ERP studies showed that N2 and P3 components were important during processing of painful stimuli, as these two components are both associated with the process of selective attention and might reflect how participants allocated attentions to more important or relevant events and screened out distractive and contradicting environmental information (Gu and Han, 2007; Sun et al., 2017; Wu et al., 2017). Temporally, empathy for pain consisted of an earlier and a later processing stage, and the later stage was more likely to be influenced by the attention shifting (Fan and Han, 2008). Recent eye-tracking research also divided attentional process into early attention orientation stage and late attention maintenance stage (Yang et al., 2012; Vervoort et al., 2013). Eye-tracking studies showed that fixation count and total fixation duration of pain-related stimuli, measures of the later stage, were associated with participants’ evaluation of pain intensity (Yan et al., 2016, 2017). Both research in ERPs and eye-tracking suggest that empathy for pain would be influenced by attention, especially processes in the late attention maintenance stage, and the current study provided findings consistent with this.

### Expected and Expressed Pain

The current study moved a step further from previous research and investigated the result of pain instead of expected pain. In order to test whether physical cue would influence children’s evaluation of pain and genuineness judgment, we present physical cue and painful facial expression at the same time and instructed participants about the casual relationship. Previous research mainly used pictures of physical injury or non-injury to induce participants empathy (Jackson et al., 2005; Shamay-Tsoory et al., 2013), but these manipulations would more likely test expected pain (Atlas et al., 2010), and were not suitable for testing genuineness judgment. In our experiment the facial expression always showed pain, but physical cue would be valid or invalid. It allows us to exam the pure causal relationship between physical cue and children’s evaluation of pain. At the same time, we need to admit the interference effect of painful facial expression. It could explain children’s high tendency to believe other’s pain is true.

## Strengths, Limitation, and Conclusion

To our knowledge, this is the first study that investigate the relationship between physical cue and children’s empathy for pain and its cognitive process. With respect to the experiment paradigms and materials, there are still some limitations. First, because fake pain is less common in daily life and it is hard to give a visual representation, we did not include an invalid condition in Experiment 2, so further research would need to consider such a condition and investigate its effect on eye-tracking. Second, children’s empathy for pain is highly positively related to their trait empathy (Yan et al., 2017), but this study did not measure trait empathy and could not separate its influence. Still, this should not be a serious problem as both experiments followed a fully within-subject design.

For future, there are some suggestions. First, previous research showed that attention was closely related to one’s executive functions (Best and Miller, 2010; Diamond, 2013), and further research will also be needed to examine whether executive functions play a role in the mechanisms underlying our findings. Second, findings would probably be different in health care professionals who always tend to underestimate other’s pain, as Kappesser et al. (2006) found that verbal report from patients would reduce professionals’ evaluation of patients’ pain intensity. Third, according to physical and social pain overlap theory (Eisenberger, 2012), pain indicated by social cue would be the same as indicated by physical cue, thus further research could consider investigating the influence of social cue to children’s empathy for pain. Sun et al. (2016) use pictorial visual-probe task to test whether verbal instruction would influence adults’ attentional bias to painful picture, and they found that the experimental group which received verbal instruction before the tasks would exhibit weaker attention bias toward painful pictures. Fourth, more types of cues could be used, and a study indeed has found that uncertain cues would induce stronger reaction to pain than certain cues (Lin et al., 2014).

In developmental sense, the current study may have two contributions: (1) it deepened our understanding of the development of empathy for pain; (2) it used eye tracking to provide evidence for a visual profile of the relationship between physical cue and children’s empathy for pain.

## Ethics Statement

All procedures performed in the study involving human participants were conducted in accordance with the ethical standards of the institutional and national research committee and with the 1964 Helsinki declaration and its later amendments or comparable ethical standards. Informed consent was obtained from all participants included in the study.

## Author Contributions

ZY and YS contributed to the conception and design of the work. ZY collected and analyzed the data. ZY, MP, and YS contributed to the writing of the manuscript.

## Conflict of Interest Statement

The authors declare that the research was conducted in the absence of any commercial or financial relationships that could be construed as a potential conflict of interest.
